# Hepatoprotective Activity of *Leptadenia hastata* (*Asclepiadaceae*) on Acetaminophen-Induced Toxicity in Mice: *In Vivo* Study and Characterization of Bioactive Compounds through Molecular Docking Approaches

**DOI:** 10.1155/2020/3807234

**Published:** 2020-08-31

**Authors:** Borris R. T. Galani, Brice A. Owona, Dieudonné P. D. Chuisseu, Esaïe Machewere, Claude B. N. Ngantchouko, Paul F. Moundipa

**Affiliations:** ^1^Laboratory of Applied Biochemistry, Department of Biological Sciences, Faculty of Science, University of Ngaoundere, P.O. Box 454 Ngaoundere, Cameroon; ^2^Laboratory of Pharmacology and Toxicology, Department of Biochemistry, Faculty of Science, University of Yaounde I, P.O. Box 812 Yaounde, Cameroon; ^3^Department of Medicine, Medical and Biomedical Sciences, Higher Institute of Health Sciences, Université des Montagnes, P.O. Box 208 Bangangte, Cameroon; ^4^Department of Pharmacy, Higher Institute of Health Sciences, Université des Montagnes, P.O. Box 208 Bangangte, Cameroon

## Abstract

**Materials and Methods:**

Various aqueous extracts were prepared from this plant and preadministered per os to albino mice 3 h before APAP administration, once daily for one week. Animals from the normal group were given only distilled water while those from negative control received only APAP 250 mg/kg. After treatment, mice were sacrificed, the liver was collected for histopathology analysis, and different biochemical markers (alanine aminotransferase (ALT), superoxide dismutase (SOD), catalase (CAT), glutathione (GSH), malondialdehyde (MDA), and tumor necrosis factor-alpha (TNF*α*)) were measured. The content of the active extract was analyzed by HPLC/UV. Molecular docking was conducted using iGEMDOCK software, and the drug-likeness and pharmacokinetic profiles were evaluated using Swiss ADME.

**Results:**

APAP administration significantly increased (*p* < 0.001) ALT in liver homogenates when compared to normal controls whereas the stem decoction at 250 mg/kg significantly (*p* < 0.001) reduced this activity to a normal value comparable to silymarin 50 mg/kg which is better than leaf and root extracts. Moreover, the stem decoction also significantly reduced the MDA levels (*p* < 0.05) and increased those of GSH, SOD, and CAT (*p* < 0.001) at doses of 250 and 500 mg/kg compared to the negative control. A significant (*p* < 0.001) decrease of TNF*α* levels and leukocyte infiltration was observed following treatment with this extract. The HPLC/UV analysis of the decoction revealed the presence of dihydroxycoumarin, quinine, and scopoletin with the following retention times: 2.6, 5.1, and 7.01 min, respectively. *In silico* studies showed that quinine and dihydroxycoumarin had great potentials to be orally administered drugs and possessed strong binding affinities with TNF*α*, TNF receptor, cyclooxygenase-2, iNOS, cytochrome P450 2E1, and GSH reductase.

**Conclusion:**

Based on these results, *L. hastata* could be considered a source of promising hepatoprotective compounds with antioxidant and anti-inflammatory properties.

## 1. Introduction

The liver is a central organ in energy metabolism and the biotransformation of xenobiotics. Therefore, frequent exposure to toxic xenobiotics is likely to provoke a liver injury, resulting in cirrhosis, liver cancer, and acute liver failure [1]. Acetaminophen (paracetamol or N-acetyl-para-aminophenol (APAP)) is one of the most widely used analgesics and antipyretic drugs worldwide. Although considered safe at therapeutic doses (up to 4000 mg/day), APAP, at higher doses, can induce centrilobular necrosis which generally leads to a fatal outcome [2]⁠. APAP intoxication would be responsible for about one-half of all cases of acute liver failure in the United States and the United Kingdom [3]⁠. In a recent study in Thailand where 184 patients with APAP overdose were included, 15.6% were reported with mild hepatotoxicity and 6.4% developed severe hepatotoxicity while 3 (1.6%) patients had acute liver failure [4]⁠.

APAP hepatotoxicity is initiated by the production of N-acetyl-p-quinone-imine (NAPQI), a reactive metabolite generated by cytochrome P450 enzymes that metabolize the drug when present at high doses. This compound then depletes glutathione stores and binds to several cellular proteins especially mitochondrial proteins, thereby leading to mitochondrial oxidant stress which causes cell death [4]⁠. Moreover, it was shown that the immune system plays a role in the progression of APAP-induced hepatotoxicity. Indeed, activated Kupffer cells produce some proinflammatory and chemotactic cytokines which promote infiltration of neutrophils and macrophages in the liver tissue, causing therefore an exacerbation of liver damage [5]⁠. To block this toxicity, coadministration of N-acetylcysteine (NAC), a cysteine-derived antidote, is often useful, but some side effects, such as hypotension, limit its efficacy [6]**⁠**. Consequently, the search for novel liver-protective agents is necessary to reinforce the existing therapeutic arsenal.

*L. hastata* is a plant of the Asclepiadaceae family found everywhere in tropical Africa. The plant is consumed as legumes and used in traditional herbal medicine to treat diabetes [7]⁠. Previous studies reported trypanocidal [8],⁠ hypoglycaemic and antidiabetic [9, 10]⁠, anti-inflammatory [11, 12]⁠, and antiandrogenic properties [13]⁠. Previous reports highlighted the presence of several phytochemicals such as saponins, proanthocyanidins, phenolic glycosides, and flavonoids in the leaf extract [14–16]⁠ but also lutein, lupeol, *β*-carotene, kidjolanin, cynanforidin, and gagaminin were previously identified [16, 17]⁠. In recent years, the hepatoprotective effects of the methanolic leaf extract have been demonstrated against alcohol-induced liver injury [18]⁠. The plant extract inhibited transaminases, total cholesterol, triglycerides, and total bilirubin in comparison with the alcohol-treated rats. Although this activity was reported, little is still known about the hepatoprotective mechanisms of *L. hastata* especially against APAP and especially how the plant active compounds interact with cell proteins to afford liver protection. Therefore, the objective of this study was to evaluate the protective effects of different aqueous extracts of *L. hastata* on APAP-induced liver damage in mice. We also analyzed the phytochemical profile of the aqueous decoction by high-performance liquid chromatography (HPLC) and conducted *in silico* studies of some of the active ingredients on anti-inflammatory and antioxidant mediators.

## 2. Materials and Methods

### 2.1. Reagents

Silymarin (140 mg tablet, purity: ≥95%) was provided by Sanofi-Aventis (Paris, France). Acetaminophen (Doliprane, 500 mg tablet) was provided by Micro Labs Limited (Karnataka, India). The ALT and TNF*α* kits used for the assays were provided by Linear Chemical S. L. Joaquim Costa 18 2a planta (Barcelona, Spain) and Solarbio Life Sciences (Beijing, China), respectively.

### 2.2. Plant Material

The stems, leaves, and roots of *L. hastata* used in this study were harvested during the rainy season (August 2018) in Touboro (North, Cameroon). After harvesting and washing with water, the samples were shade-dried for two weeks, then pulverized in a mortar, and the obtained powder was sieved through a 0.5 mm mesh sieve. The botanical identification of the plant was made by Prof. Mapongmetsem Pierre-Marie, a botanist at the Faculty of Science of the University of Ngaoundere, Cameroon.

### 2.3. Preparation of Plant Extracts

For the different extract preparations, three methods of preparation, namely, infusion, decoction, and maceration were used. Twenty-five (25) g of the *L. hastata* powder was weighed and mixed with 250 mL of distilled water. For the decoction, the mixture was boiled at 100°C for 20 minutes. For the infusate, the powder was mixed with boiling water for 20 min, while for macerate, the powder was mixed with distilled water for 20 min at room temperature. After cooling, the mixtures were filtrated and the filtrates were frozen and lyophilized at -55°C. The freeze-dried extracts were packed in aluminum foil and stored in boxes at 4°C until use. Finally, test samples were solubilized in distilled water before giving to animals at the desired doses.

### 2.4. High-Performance Liquid Chromatography Analysis

To identify the components of the stem aqueous decoction of *L. hastata*, an HPLC analysis was carried out on an Agilent 1260 Infinity brand chromatographic chain using a reversed-phase C18 Inerstil-ODS-3 column (150 × 4.6 mm, 5 *μ*m, GL Sciences Inc., Tokyo, Japan) and equipped with a binary pump, autosampler, column oven, and a diode array detector from Agilent 1100 series, as previously described [19]⁠. Twenty microliters of extract dissolved in methanol was injected into the column, and the column injector was kept at 35°C during the whole chromatography. The mobile phase consisted of phosphoric acid (H_3_PO_4_), pH 2.5 (A), and acetonitrile (B). The gradient elution system was programmed as follows: 77% A and 23% B, 0-10 min at a flow rate of 0.7 mL/min; 55% A and 45% B, 11-20 min at a flow rate of 0.7 mL/min; 35% A and 65% B, 21-27 min, 5% A and 95% B, 28-31 min; and 77% A and 23% B, 32-35 min at a flow rate of 0.9 mL/min. The detection wavelength of the constituents was 320 nm. Compounds were identified based on the retention time and absorbance spectra of pure standards.

### 2.5. Experimental Animals and Treatments

Nine-week old, male and female albino mice (*Mus musculus*) weighing between 18 and 31 g were purchased from the pet store of the National School of Agro-Industrial Sciences (NSAIS) of the University of Ngaoundere. Animals were housed in plastic cages containing chips. Mice were used according to the Animal Research: Reporting of In Vivo Experiments (ARRIVE) guidelines, and ethical clearance was granted by the Animal Ethics Committee of the Faculty of Science of the University of Ngaoundere (ethical clearance number 201/2019/UN/DFS/VD-RC/CSPDR). Mice were acclimatized for 2 weeks to the lab conditions before the experiments started. Previous studies showed that the leaf aqueous extracts have a nontoxic effect in mice up to a dose of 4000 mg/kg body weight [20]⁠. Additionally, hepatoprotective effects were recently demonstrated with leaf extracts using 250 and 500 mg/kg doses [18]⁠. Consequently, we investigated the effects of aqueous leaf extracts using these doses.

In order to evaluate the effect of extracts from different plant parts on APAP toxicity, 30 mice were segregated into 6 groups of 5 mice each, presented as follows: group I—normal control was provided with 10 mL/kg distilled water; group II—negative control was treated with 250 mg/kg APAP; group III—positive control received 250 mg/kg APAP and 50 mg/kg silymarin; and groups IV, V, and VI—these groups received 250 mg/kg of APAP and 250 mg/kg of the decoction of leaves, stems, and roots, respectively. Hepatotoxicity was induced by administering 250 mg/kg of APAP to all mice, except the normal controls which only received distilled water. The plant extract and silymarin were given orally 3 h after APAP treatment and once daily for one week.

At the end of this first experiment, different aqueous extracts (decoction, macerate, and infusion) were prepared from the most active plant part, and the hepatoprotective effects were assessed. Mice were grouped and treated as mentioned above. However, in groups IV, V, and VI, the animals received 250 mg/kg of APAP and 250 mg/kg of the decoction or macerate or infusion of the most effective plant part.

Treatments lasted 7 days, and on the 8^th^ day, animals were sacrificed and the livers were collected, crushed and then centrifuged at 4000 rpm for 10 minutes at 4°C. Afterward, alanine aminotransferase (ALT) levels were then measured in liver samples. At the end of this test, the method of extraction with the most effective hepatoprotective activity was retained for further investigations at different concentrations of the plant extract. Seven groups of 5 mice each were used in this test as follows: groups I, II, and III were treated as mentioned above while Group IV, V and VI received 250 mg/kg of APAP and 100, 250, or 500 mg/kg of the stem decoction, respectively. Group VII received only 500 mg/kg of stem decoction daily for one week. At the end of treatment, mice were fasted for 12 h and thereafter sacrificed by cervical dislocation. The liver was collected, rinsed with a physiological saline solution, and weighed.

### 2.6. Biochemical Analysis

Liver ALT, superoxide dismutase (SOD), catalase (CAT), malondialdehyde (MDA), and glutathione (GSH) levels were determined using commercially available kits following the manufacturer's instructions. Briefly, the livers were homogenized in nine volumes of ice-cold 0.1 M phosphate buffer (pH 7.4). The homogenate was centrifuged at 10,000× g for 15 min at -4°C, and aliquots of the supernatants were collected for the quantification of biochemical parameters. ALT, SOD, CAT, MDA, and GSH were measured by colorimetric assays while tumor necrosis factor-alpha (TNF*α*) levels were determined by sandwich ELISA using a commercial kit from Solarbio Life Sciences (Beijing, China).

### 2.7. Histopathological Examination

The liver tissue was fixed in 4% paraformaldehyde, embedded in paraffin, sectioned into 5 *μ*m thickness, and stained with hematoxylin (H&E) for the evaluation of histopathological changes. Microscopic observations were done under a bright-field microscope. Different pathological grades were identified to evaluate the severity of inflammation: grade 0 (no pathological change), grade 1 (minimal leukocyte infiltration), grade 2 (mild), grade 3 (moderate), and grade 4 (important leukocyte infiltration) [20, 21].

### 2.8. In Silico Molecular Docking

**Figure 1:**
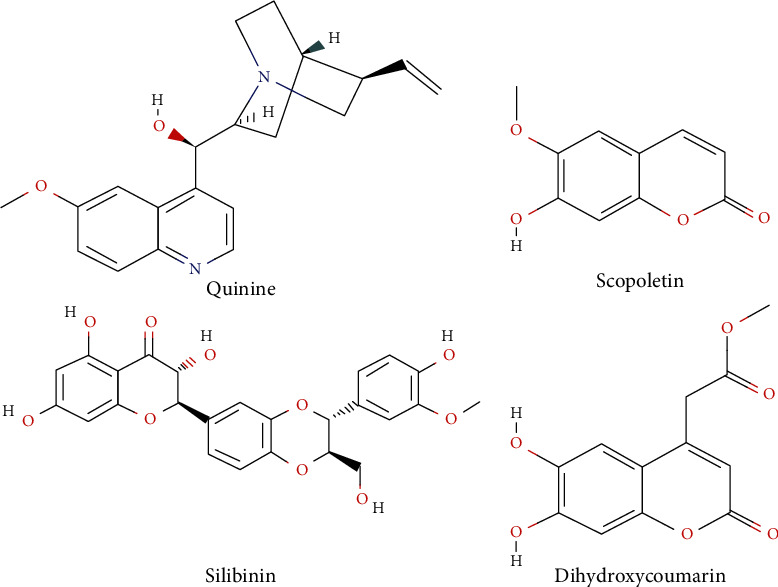
2D structures of the different docked compounds.

The phytochemical analysis of *L. hastata* aqueous extract by HPLC detected the presence of some compounds such as quinine, scopoletin, and dihydroxycoumarin. These compounds (structures shown in Figure 1) were selected to evaluate the possible interactions with target proteins involved in the inflammation and the oxidative stress during APAP toxicity in mice. This allowed us to better analyze and understand the molecular mechanisms underlying the hepatoprotective activity of this plant. Docking studies were performed using iGEMDOCK version 2.1, a graphical automatic drug design system for docking, screening, and analysis provided by BioXGEM Lab.

#### 2.8.1. Ligand Preparation

The 3D structures of the three test compounds and silibinin used as reference compound were retrieved from PubChem database in SDF format (CIDs: 57362743 (dihydroxycoumarin), 3034034 (quinine), 5280460 (scopoletin), and 31553 (silibinin)) and then converted into MDL MOL files using Open Babel. The 3D structures of proteins were downloaded from the protein data bank (PDB). These are as follows: TNF*α* (PDB ID: 6MKB), TNF receptor (TNFR) (PDB ID: 1XU2), iNOS (PBD ID: 2ORQ), cyclooxygenase-2 (Cox-2; PBD ID: 5JVY), GSH reductase (PDB ID: 2LV3), and cytochrome P450 2E1. Since the 3D crystal structure of *Mus musculus* cytochrome P450 2E1 was not present in the PDB repository, its FASTA sequence (ID: Q05421) was obtained from Uniprot and a homology modeling was performed using the SWISS-MODEL program.

The ligands were docked with PDB file's binding sites using an exact docking feature (standard docking). The following parameters were applied: population size = 200, generation = 70, and number of solutions = 2. The postanalytic method permitted us to visualize and categorize the evaluated compounds by combining drug interactions and energy-based measurement function.

### 2.9. ADME and Drug-Likeness Evaluation

For a compound to be considered a good drug candidate, its physicochemical properties must determine great chances of bioavailability. A means to verify this is to assess the drug-likeness profile of the molecule using Lipinski, Ghose, Veber, Egan, and Muegge's rules before any *in vivo* study. These rules help to predict the pharmacokinetic profile of the compound in the human body, notably absorption, distribution, metabolism, and excretion (ADME) [22]⁠. In our study, we evaluated the drug-likeness features of the 4 docked compounds using calculations based on the abovementioned rules. The ADME parameters such as gastrointestinal absorption (GI), Blood-Brain Barrier (BBB), P-glycoprotein- (Pgp-) mediated efflux, and the capacity to be used as substrates of cytochrome P450 (CYP) were also analyzed using SwissADME, a free web tool available at http://www.swissadme.ch/.

### 2.10. Statistical Analysis

All data were expressed as the mean ± standard error to mean (SEM). Statistical analysis was carried out using the GraphPad Prism 5.0 software for windows. The comparison of means was performed using a one-way analysis of variance (ANOVA) followed by Dunnett's post hoc tests. Differences were considered significant when *p* < 0.05.

## 3. Results

### 3.1. L. hastata Significantly Improved Liver ALT Activities in APAP-Treated Mice

**Figure 2:**
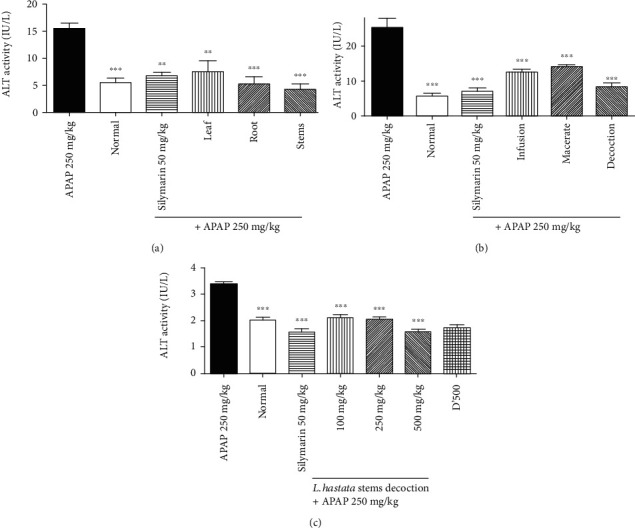
Effect of *L. hastata* aqueous extracts on liver ALT activity in APAP-induced hepatotoxicity. (a) The effect of leaf, root, and stems. (b) The effect of the infusion, macerate, and decoction of stems. (c) The effect of different doses of stem decoction. The error bars represent the SEM of 5 mice in six separate groups (*n* = 30).

The quantification of ALT in liver homogenates has been previously reported to be a reliable marker of liver injury [20, 22]⁠⁠⁠.  Figure 2 shows the effect of *L. hastata* on the ALT activity in different experimental groups.

Our findings indicate that ALT activity was significantly (*p* < 0.001) higher in the APAP-treated group (15.5 ± 1.01 IU/L) as compared to the normal group (5.5 ± 0.86 IU/L). However, silymarin and the leaf, root, and stem aqueous extracts significantly decreased this activity from 15.5 ± 1.01 IU/L to 6.75 ± 0.65 IU/L, 7.5 ± 2.02 IU/L, 5.25 ± 1.29 IU/L, and 4.25 ± 1.01 IU/L, respectively, (Figure 2(a)). In another experiment, we found that the posttreatment of mice with the stem decoction reduced by 67.32% (from 25.25 ± 2.81 IU/L to 8.25 ± 1.08 IU/L) of the liver ALT activity, less than silymarin (72.95%, 6.83 ± 1.08 IU/L) and better than infusion (50.49%, 12.5 ± 0.86 IU/L) and macerate (44.55%, 14.00 ± 0.57 IU/L) (Figure 2(b)). Further analysis demonstrated that this effect was dose-dependent (Figure 2(c)) while no toxic effect was observed at 500 mg/kg.

### 3.2. The Stem Decoction of *L. hastata* Modulated the Levels of Oxidative Parameters in the Liver of APAP-Treated Mice

Oxidative stress is an important phenomenon in APAP pathogenesis. To evaluate the capacity of our plant extracts to regulate this process, we measured the effect of different doses of the decoction of *L. hastata* stems on MDA and GSH levels, as well as on CAT and SOD activities in each batch of mice after APAP poisoning.

**Figure 3:**
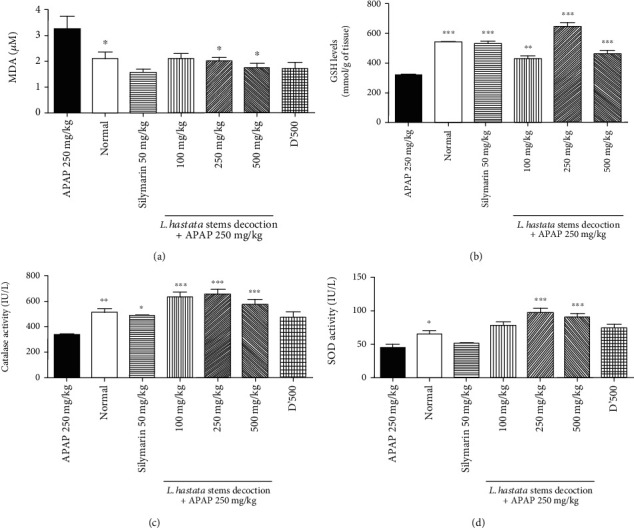
Effect of different doses of the *L. hastata* stem decoction on oxidative stress parameters in liver homogenates. (a) MDA levels. (b) GSH levels. (c) Catalase activity. (d) SOD activity. D'500 represents a group of mice treated only with plant extract at 500 mg/kg without APAP administration. The error bars represent the SEM of 5 mice in six separate groups (*n* = 30).

The results in Figure 3 show that administration of APAP (250 mg/kg) significantly increased (Figure 3(a), *p* < 0.05) MDA levels from 2.12 ± 0.23 *μ*M to 3.26 ± 0.49 *μ*M and decreased the GSH levels (Figure 3(b), *p* < 0.01) from 542.9 ± 2.38 mmol/g of liver tissue to 321.2 ± 2.82 mmol/g of liver tissue, the CAT activity (Figure 3(c), *p* < 0.01) from 510.9 ± 27.4 IU/L to 338.4 ± 11.74 IU/L, and the SOD activity (Figure 3(d), *p* < 0.05) from 65.34 ± 5.06 IU/L to 45.43 ± 4.09 IU/L compared to the normal control. However, *L. hastata* decoction significantly improved these parameters at the different tested doses.

### 3.3. Stem Decoction of *L. hastata* Counteracted Liver Inflammation in APAP Toxicity by Downregulating TNF*α* Expression and Decreasing Leukocyte Infiltration

**Figure 4:**
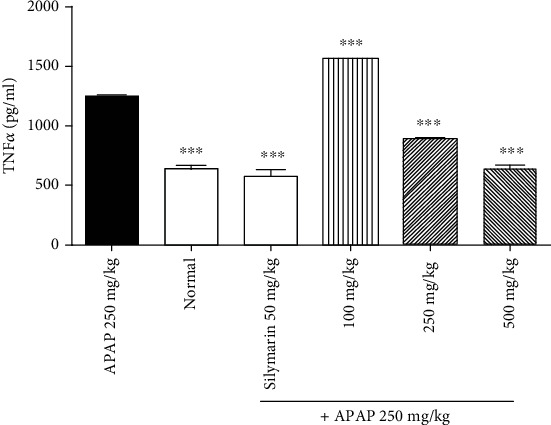
Dose-dependent effect of the *L. hastata* stem decoction on TNF*α* levels in liver homogenates of APAP-treated mice. The error bars represent the standard error to mean of 5 mice in six separate groups (*n* = 30).

Administration of APAP (250 mg/kg) to mice induced an increased expression of TNF*α* protein in liver homogenates by 1.95-fold as compared to normal control (Figure 4). However, treatment with the stem decoction showed a significant dose-dependent decrease of TNF*α* with the 500 mg/kg dose standing out as the one with the better activity. Stem decoction at this dose decreased the TNF*α* concentration from 1250 ± 16.67 pg/mL to 633.3 ± 34.69 pg/mL similarly as silymarin (566.7 ± 66.67 pg/mL) as compared with the APAP-treated group.

**Figure 5:**
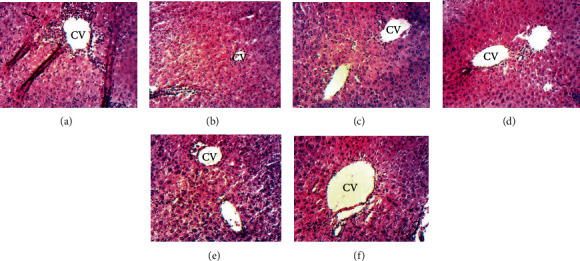
Micrographs of liver sections of mice treated with different doses of the *L. hastata* stem decoction (magnification ×100). (a) APAP-treated mice (negative control). Black arrows show high leukocyte infiltration around the centrilobular vein (CV); (b) normal control; positive control—(c) 250 mg/kg APAP+silymarin (50 mg/kg), (d) 250 mg/kg APAP+*L. hastata* stem decoction (100 mg/kg), (e) 250 mg/kg APAP+L. hastata stem decoction (250 mg/kg), and (f) 250 mg/kg APAP+L. hastata stem decoction (500 mg/kg).

The effect of *L. hastata* decoction was also examined through hematoxylin/eosin staining to confirm the necroinflammatory activity found in the liver tissue. As shown in Figure 5(a), APAP administration led to significant neutrophil infiltration around the portal vein, with hepatic necrosis compared to the normal group (Figure 5(b)) where no pathological changes were observed. The plant decoction at 100 and 500 mg/kg decreased considerably the pathological score as compared to the APAP group. This decrease was similar to that of silymarin at 50 mg/kg (Table 1 and Figures 5(c)–5(f)). Nonetheless, with the plant extract at 250 mg/kg, a partial restoration of the liver structure was observed.

### 3.4. HPLC Profile of the Stem Decoction of *L. Hastata*

**Figure 6:**
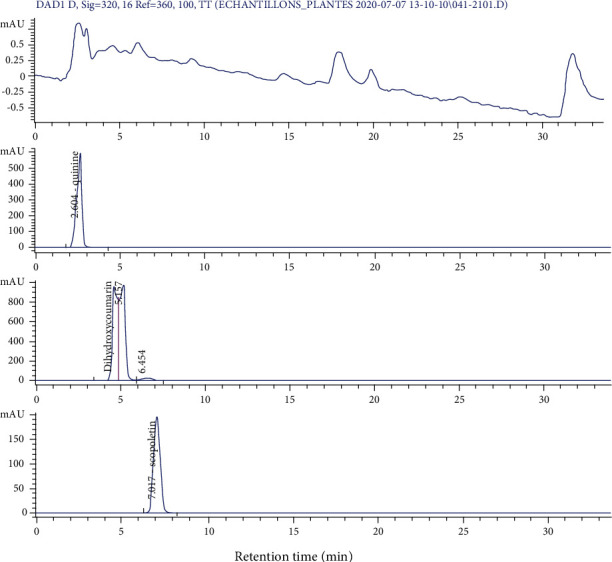
HPLC/UV chromatogram of the stem decoction of *L. hastata* in comparison with those of quinine, dihydroxycoumarin, and scopoletin at 320 nm.

Figure 6 shows the HPLC-UV chromatogram of the aqueous decoction of *L. hastata* at 320 nm in comparison with those of the standard compounds used.

It appears from this figure that the plant extract contains several compounds, some of which have retention times corresponding to quinine (2.60 min), dihydroxycoumarin (5.51 min), and scopoletin (7.01 min). These compounds were therefore used for *in silico* studies.

### 3.5. In Silico Screening Data

**Figure 7:**
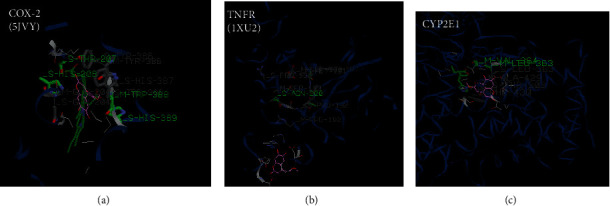
Docking pose of dihydroxycoumarin with (a) Cox-2 (PDB ID: 5JVY), (b) TNFR (PDB ID: 1WU2), and (c) CYP 2E1 (3D structure modelized by a Swiss model). Dihydroxycoumarin structure is colored in pink.

**Figure 8:**
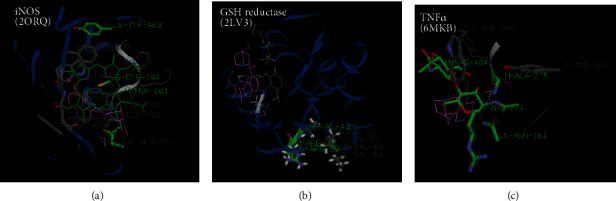
Docking pose of quinine with (a) iNOS (PDB ID: 2ORQ), (b) GSH reductase (PDB ID: 2LV3), and (c) TNF*α* (PDB ID: 6MKB). Quinine structure is colored in pink.

**Figure 9:**
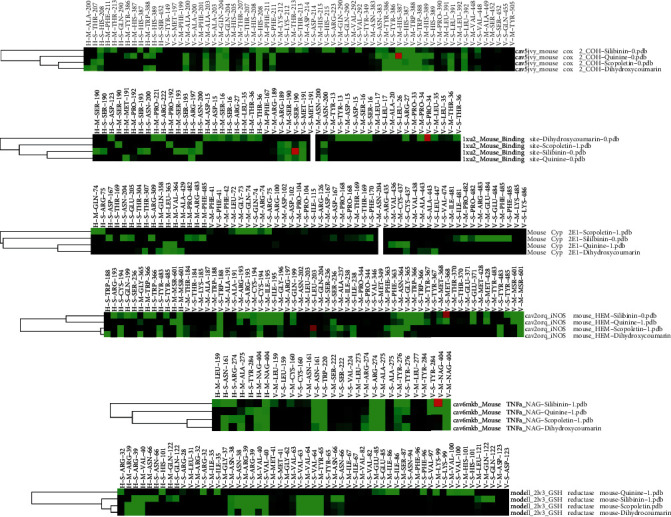
Interaction profile clusters of docked compounds with iNOS, TNF*α*, GSH reductase, Cox-2, TNFR, and CYP 2E1. H and V indicate hydrogen bonding and van der Waals interactions, respectively, while M and S correspond to the main chain or side chain of interacting residues. The interactions are colored in green when the energy is ≤1.5.

From the docking results, higher binding energies were particularly found with dihydroxycoumarin and quinine than scopoletin during the interaction with the different PDB files. However, these binding energies remained lower compared to silibinin, used here as a reference compound. As shown by Table 2, dihydroxycoumarin showed a higher binding affinity for COX-2 (-101.3 kcal/mol), TNFR (-110.86 kcal/mol), and CYP 2E1 (-103.45 kcal/mol) than quinine and scopoletin, while quinine exhibited better fitness values with iNOS (-119.63 kcal/mol), TNF*α* (-68.81 kcal/mol), and GSH reductase (-98.18 kcal/mol) compared to dihydroxycoumarin and scopoletin. However, the score obtained with dihydroxycoumarin was not far from that of quinine for the iNOS protein. A deep analysis of the interactions profiles reveals that the binding of these compounds included both hydrophobic and hydrogen bonds. The docked poses of the dihydroxycoumarin showed strong hydrogen bonds with Ala 200, Thr 207, Trp 388, and His 389 in the binding site of Cox-2 (Figure 7(a)), while Asp 15, Ser 16, Arg 27, Thr 36, Asp 123, Pro 221, and Arg 222 were involved in hydrogen bonding with the TNFR's binding site (Figure 7(b)). As far as CYP 2E1 is concerned, strong hydrogen bindings were established with Thr 307, Leu 363, Val 364, and Ala 429 in the enzyme binding site (Figure 7(c)). Concerning quinine, hydrogen bonds were formed with MSR 601 in the interaction with iNOS (Figure 8(a)), Arg 32 and His 101 in the interaction with GSH reductase (Figure 8(b)), and Ala 275, Tyr 284, and NAG 404 in the interaction with TNF*α* (Figure 8(c)). Also, quinine presented a binding profile closer to silibinin as far as TNF*α*, iNOS, and GSH reductase are concerned. For dihydroxycoumarin, its binding mechanism on TNFR is more different from other compounds and closer to scopoletin for COX-2 (Figure 9).

### 3.6. Drug-Likeness and Pharmacokinetic Profile of the Docked Compounds

The predicted pharmacokinetic and drug-likeness profile of the different docked compounds is shown in Table 3. Except for silibinin, quinine, dihydroxycoumarin, and scopoletin have a high GI absorption and complied with almost all the drug-likeness rules including the Lipinski filter which proves their potential to be used as oral drugs. Besides, all docked compounds showed a good bioavailability score of 55%. However, silibinin and dihydroxycoumarin were found unable to cross the BBB. Furthermore, none of these compounds stood out as a substrate of permeability glycoprotein (P-gp), a transporter that permits to estimate active efflux through biological membranes. Concerning the skin permeation, the more negative the log Kp, the less skin permeant is the molecule. According to our data, quinine has the greatest chances to cross skin with a log Kp of -6.23 cm/s followed by scopoletin, dihydroxycoumarin, and silibinin. CYP p450 enzymes play an important role in drug elimination, through metabolic biotransformation. It was suggested that the inhibition of these enzymes is one of the major causes of drug-drug interactions leading to toxic or undesirable effects [22]⁠. Quinine showed selectivity for CYP 2D6 while scopoletin and silibinin showed their selectivities for CYP 1A2 and CYP 3A4, respectively, suggesting their capacity to cause drug interactions. However, dihydroxycoumarin was found unable to cause such inhibition with all the p450 isoforms tested.

## 4. Discussion

APAP-induced hepatotoxicity especially in mice is an attractive and clinically relevant model that is widely used to assess the efficacy of hepatoprotective natural products. Indeed, similarities were reported with mechanisms of APAP toxicity in human cells. In this model, liver injury usually appears after 3–5 h and peaks at 12 h with doses of 200–400 mg/kg APAP, similar to human overdoses, which varied from 150 to 500 mg/kg [23]. In this study, we tested the hepatoprotective potential of aqueous extracts of *L. hastata* on APAP-induced liver injury in mice. Our results showed that APAP administration (250 mg/kg) led to a significant (*p* < 0.01) increase in liver ALT levels compared to normal control. APAP also induced a significant increase in MDA (*p* < 0.05) and TNF*α* (*p* < 0.001) levels compared to the normal control. Conversely, it induced a very significant decrease in the activity of CAT, SOD, and GSH compared to the normal control group. These observations would be due to the accumulation of NAPQI which would lead to GSH depletion, thereby promoting the formation of reactive oxygen species [23, 24]⁠. The results obtained are similar to those reported by Hu et al. who showed that the administration of APAP at a dose of 250 mg/kg was sufficient to induce hepatotoxicity with hemorrhagic lesions and increased hepatic MDA levels and decreased GSH values [25]⁠. The increase in inflammation is thought to be due to the formation of NAPQI-cysteine protein adducts [26]⁠ and MDA-lysine which are presented to cytotoxic T cells by antigens. This causes a cascade of reactions that result in the activation of Kupffer cells, allowing the secretion of inflammatory mediators, cytokines, and chemokines that promote hepatocyte apoptosis and activate the innate immune system [1, 3]**⁠**. These results are also in concordance with earlier reports which showed that the hepatoprotective effect of tempol is mediated by an overexpression of antioxidant enzymes in APAP-treated mice [27]⁠. Therefore, activation of these enzymes might suggest induction of the nuclear factor erythroid 2-related factor (Nrf-2) pathway.

Furthermore, we also found that stem decoction of *L. hastata* was able to reverse all these biochemical and histopathological changes observed in APAP toxicity. These effects may be attributed to the active metabolites contained in this extract which can interfere with the inflammatory process and the oxidative stress induced by APAP. These findings are in line with previous studies that demonstrated the protective effect of *L. hastata* on alcohol-induced damages [18] and the effect of *L. reticulata* against carbon tetrachloride-induced toxicity [28]⁠. Although we found that the hepatoprotective effects of *L. hastata* were mediated by inhibition of the oxidative stress and inflammation in the liver, further biochemical markers should be explored for a better understanding of the mechanism of action. In particular, the effect on anti-inflammatory and other proinflammatory cytokines, P450 enzyme expression, Nrf2 activation, apoptosis, and autophagy processes should be investigated. Also, the effect of this plant on liver regeneration was not yet evaluated although it was considered a critical aspect of APAP's pathophysiology [29]. Such studies must be conducted using isolated phytochemicals from *L. hastata*.

To characterize the constituents of the active extract of *L. hastata*, an HPLC analysis was performed and results revealed the presence of many compounds including dihydroxycoumarin, scopoletin, and quinine, a natural alkaloid known for its antipyretic, analgesic, and hepatoprotective properties in malaria patients [30]⁠. These results are consistent with previous phytochemical studies which reported the presence of alkaloids and coumarins in *Leptadenia hastata* [31]⁠.

In order to better examine the role of these compounds in the hepatoprotective activity against APAP, their binding affinities with some antioxidant and proinflammatory mediators were analyzed by molecular docking using the iGEMDOCK software. The binding energy, van der Waals interaction, electrostatic energy, and hydrogen bonding were calculated to determine how the ligand binds to the target protein and visualize the pharmacological interactions. As shown by Guimarães et al., negative *Δ*G binding values indicate favorable interactions of the ligand-receptor complex [32]⁠. The docking interactions presented in Table 2 demonstrated that dihydroxycoumarin had the lowest binding energies with COX-2 (-101.3 kcal/mol), CYP 2E1 (-103.45 kcal/mol), and TNFR (-110.86 kcal/mol), while quinine presented the strongest affinity with GSH reductase (-98.18 kcal/mol), TNF*α* (-68.81 kcal/mol), and iNOS (-119.63 kcal/mol).

COX-2 are key enzymes involved in prostaglandin biosynthesis in inflammatory cells. It was previously showed that the COX-2 active site possesses three important regions including a hydrophobic pocket identified by the presence of Tyr 385, Trp 387, Phe 518, Ala 201, Tyr248, and Leu 352, a second key region associated with three hydrophilic amino acid residues (Arg 120, Glu 524, and Tyr355) located at the entrance of the active site and a side pocket characterized by the presence of His 90, Arg 513, and Val 523 [33]⁠. On contrary to the reported data, our docking results seem to indicate that the COX-2-dihydroxycoumarin complex involves interactions at a position different from the active site. Indeed, as shown in Figure 9, hydrogen bonds were formed with Ala 200, Thr 207, His 208, Trp 388, and His 389 and hydrophobic interactions with Ala 200, Tyr 386, and Trp 388.

Nitric oxide (NO) is a highly reactive oxidant produced in the liver tissue in response to the proinflammatory mediators. This metabolite is generated by a family of enzymes called *inducible NO synthase* or iNOS from the L-arginine metabolism. The role of NO in APAP-induced hepatotoxicity has been demonstrated in a previous study. NO levels were found to increase in hepatocytes isolated from APAP-treated rats due to iNOS overexpression [34]⁠. The molecular docking analysis of the iNOS enzyme (PDB ID: 2ORQ) with our ligands showed high binding affinities with quinine (-119.63 kcal/mol). The interaction between quinine and iNOS in the binding cavity involved a mechanism closer to that of silibinin (Figure 9). These results are similar to those reported by Chen et al. (2005) who demonstrated the inducible effect of chloroquine on human iNOS [35]⁠.

TNF*α* is a cytokine released by activated macrophages (Kupffer cells) in the liver in response to toxic agents like APAP. TNF*α* has been linked to increased oxidative stress (increased formation of reactive oxygen species and reactive nitrogen species) and is known to recruit and activate other inflammatory cells [2]. According to our results, quinine exhibited the best binding affinity with TNF*α*. This interaction is favored by the formation of hydrogen bonds with Ala 275, Tyr 284, and NAG 404 residues and hydrophobic bonds with Asn 161, Trp 220, Arg 274, Ala 275, Tyr 276, Tyr 284, and NAG 404 residues. Previous studies showed that some quinoline derivatives with anti-inflammatory properties might form a stable complex with TNF*α* by docking in the middle pocket of dimers [36]⁠. However, no hydrogen bond was observed in these studies which could be explained by the different nature of the docked molecule and the docking approach. Furthermore, we also found a strong affinity between quinine, dihydroxycoumarin, and the TNFR as shown in Table 2. But this affinity was lower than that observed with silibinin (-123.1 kcal/mol). These findings are consistent with previous reports who identified novel inhibitors for TNF*α* and TNF*α* receptors using pharmacophore-based approaches [37]⁠. According to Chen et al. (2017), small molecules that inhibit TNF*α* or its receptor, are equally capable to block the interactions between both proteins and/or regulate related signaling pathways [38]⁠.

CYP2E1 is a cytochrome P450 enzyme implicated in the formation of NAPQI, the reactive intermediate of acetaminophen in studies in human liver microsomes [39]. Previous studies showed that the downregulation of CYP 2E1 by hepatoprotective compounds is a key mechanism of the alleviation of APAP-induced liver injury [40]. Our data also proved that dihydroxycoumarin has a great binding affinity for this enzyme. Docking studies conducted to understand the interactions of coumarins with P450 revealed that their binding modes change depending on the nature and position of substituents and the P450 enzyme binding cavity topography [41]⁠. According to these studies, one of the key interactions demonstrated by coumarins is the *π*-*π* stacking interactions with the phenylalanine residues in the binding pocket. Additionally, the hydrogen interactions determine the orientation of coumarins in the binding pocket [42]⁠. In our study, dihydroxycoumarin formed hydrogen bonds with Thr 307, Leu 363, Val 364, and Ala 429 and hydrophobic interactions with Thr 303, Thr 307, Gln 358, Leu 363, Ala, and 429 and especially with Phe 430, which support previous findings.

GSH reductase is an antioxidant enzyme involved in the regeneration of cellular GSH. The activity of this enzyme is particularly inhibited during APAP toxicity [43]⁠. Our data revealed that among the tested compounds, quinine showed the greatest binding affinity for this enzyme, even though it is still lower than the affinity of silibinin. We also noted that the GSH reductase-quinine complex is stabilized by hydrogen bonds with Arg 32 and His 101 and van der Waals interactions with Arg 32, Ile 35, Ile 86, Phe 96, Val 100, and His 101 in the active site. This result suggests that the quinine would be highly associated with the regeneration of the GSH content through GSH reductase activation. Earlier molecular docking studies on trypanothione reductase and GSH reductase demonstrated that aryloxy-quinones might exhibit a high affinity for these enzymes using a noncompetitive binding mechanism in a subsite in the catalytic one [44]. In the case of GSH reductase, the list of contacts in this study included some residues like Lys 67, Ile 113, His 467, or Glu 473. Similar amino acids were found in our study although they are located at different positions. These differences may be explained by the nature of the docked molecule and the crystal structure of glutathione reductase.

## 5. Conclusion

This study revealed that *L. hastata* stem decoction protected mice from APAP-induced hepatotoxicity through antioxidant and anti-inflammatory activities in the liver tissue. The HPLC profile of this extract revealed the presence of quinine (an alkaloid), dihydroxycoumarin, and scopoletin. The *in silico* analysis demonstrated that quinine had great binding affinities for iNOS, TNF*α*, and GSH reductase while dihydroxycoumarin fitted perfectly with Cox-2, TNFR, and CYP 2E1. Furthermore, both compounds showed good drug-likeness and pharmacokinetic scores, suggesting their potentials to be highly bioavailable oral drugs. The interactions of these compounds with the target proteins seem to be important factors in providing liver protection and might contribute to valorize the potentialities of this plant against liver diseases. Further investigations with the isolated compounds are therefore necessary to have complete information on the biological activity on the liver.

## Figures and Tables

**Table 1:** Effects of *L. hastata* aqueous decoction on the histopathological grade values induced by chronic APAP administration.

Group	*N*	Histopathological score
APAP	5	3.5 ± 0.6^≠≠^
Normal	5	0.0 ± 0.0
APAP 250 mg/kg+silymarin at 50 mg/kg	5	0.5 ± 0.2∗∗
APAP 250 mg/kg+*L. hastata* at 100 mg/kg	5	0.6 ± 0.2∗∗
APAP 250 mg/kg+*L. hastata* at 250 mg/kg	5	1.5 ± 0.4∗
APAP 250 mg/kg+*L. hastata* at 500 mg/kg	5	0.8 ± 0.4∗

≠≠ indicates significant difference (*p* < 0.01) when compared to normal control and ∗ or ∗∗ indicates significant values at *p* < 0.05 and *p* < 0.01, respectively, when compared to the APAP group.

**Table 2:** Total energy, van der Waals interaction (VDW), hydrogen bonding (Hbond), and Electrostatic energy of tested compounds on interaction with PDB files of target proteins.

#ligand	Total energy (kcal/mol)	VDW (kcal/mol)	Hbond (kcal/mol)	Elec (kcal/mol)
iNOS (PDB ID: 2ORQ)+				
Dihydroxycoumarin	-119.35	-92.42	-26.93	0
Quinine	**-119.63**	-102.45	-17.17	0
Scopoletin	-94.82	-79.07	-15.75	0
Silibinin	-127.29	-96.73	-26.14	0
COX-2 (PDB ID: 5JVY)+				
Dihydroxycoumarin	**-101.13**	-85.94	-15.2	0
Quinine	-94.62	-80.57	-14.05	0
Scopoletin	-82.44	-74.23	-8.2	0
Silibinin	-126.7	-102.32	-24.37	0
TNF*α* (PDB ID: 6MKB)+				
Dihydroxycoumarin	-67.97	-39.68	-28.29	0
Quinine	**-68.81**	-58.13	-10.67	0
Scopoletin	-56.35	-35.69	-20.66	0
Silibinin	-90.21	-61.03	-29.18	0
Mouse CYP 2E1+				
Dihydroxycoumarin	-**103.45**	-86.54	-16.9	0
Quinine	-97.41	-90.41	-7	0
Scopoletin	-81.34	-64.32	-17.02	0
Silibinin	-120.96	-88.26	-32.71	0
GSH reductase (PDB ID: 2LV3)+				
Dihydroxycoumarin	-89.16	-71.58	-17; 58	0
Quinine	**-98.18**	-92.18	-6	0
Scopoletin	-90.86	-71; 16	-19.7	0
Silibinin	-124.43	-101.58	-22.72	0
TNF*α* receptor (PDB ID: 1XU2)+				
Dihydroxycoumarin	**-110.86**	-64.9	-45.95	0
Quinine	-100.51	-90.52	-9.99	0
Scopoletin	-81.08	-66. 27	-14.81	0
Silibinin	-123.1	-90.24	-32.86	0

**Table 3:** Calculated ADME, pharmacokinetic parameters, and drug-likeness parameters for the docked compounds.

	Pharmacokinetic parameters									Drug-likeness parameters (number of violations)					
	GI absorption	BBB permeant	P-gp substrate	Cyp P450 inhibitors					Skin permeation (log Kp)	Lipinski	Ghose	Veber	Egan	Muegge	Bioavailability score
				Cyp 1A2	Cyp 2C19	Cyp 2C9	Cyp 2D6	Cyp 3A4							
Quinine	High	Yes	No	No	No	No	Yes	No	-6.23 cm/s	Yes (0)	Yes	Yes	Yes	Yes	0.55
Dihydroxycoumarin	High	No	No	No	No	No	No	No	-7.44 cm/s	Yes (0)	Yes	Yes	Yes	Yes	0.55
Scopoletin	High	Yes	No	Yes	No	No	No	No	-6.39 cm/s	Yes (0)	Yes	Yes	Yes	No (1)	0.55
Silibinin	Low	No	No	No	No	No	No	Yes	-7.89 cm/s	Yes (0)	No (1)	No (1)	No (1)	No (1)	0.55

## Data Availability

All data are available in the manuscript.
